# Functional ultrasound imaging in preclinical brain stimulation: from multimodal readouts to closed-loop neuromodulation

**DOI:** 10.3389/fnins.2026.1897417

**Published:** 2026-07-31

**Authors:** Muhang He, Leena Usman, Hamid Charkhkar, Rui Cao

**Affiliations:** 1 Department of Biomedical Engineering, Case School of Engineering, School of Medicine, Case Western Reserve University, Cleveland, OH, United States; 2 Louis Stokes Cleveland Veterans Affairs Medical Center, Cleveland, OH, United States; 3 Case Comprehensive Cancer Center, Case Western Reserve University, Cleveland, OH, United States

**Keywords:** brain–machine interface, deep brain stimulation, focused ultrasound, functional ultrasound (fUS), optogenetics

## Abstract

Understanding how brain stimulation engages neural circuits requires readouts that combine rapid monitoring, access to deep structures, and high spatiotemporal resolution. Few existing modalities can provide this combination. This minireview summarizes recent advances in the application of functional ultrasound (fUS) imaging to preclinical brain stimulation, with an emphasis on its value as an advanced functional imaging modality across different stimulation paradigms. Building on ultrafast ultrasound, fUS captures cerebral hemodynamic changes associated with neural activity, enabling near-real-time hemodynamic monitoring with high spatiotemporal resolution. We survey the applications of fUS across optogenetic stimulation, deep brain stimulation (DBS), and focused ultrasound (FUS) stimulation, covering its role in mapping stimulation-evoked network responses and characterizing parameter-dependent neuromodulation effects. We further argue that the unique compatibility between fUS as a readout and FUS as an effector points toward a fully integrated read–write closed-loop neuromodulation framework, in which fUS continuously monitors brain hemodynamic states and feeds these signals back into an adaptive controller to deliver FUS. Together, these directions position fUS as an effective and promising tool for examining the mechanisms of brain function and its responses to stimuli and for developing precise, adaptive neuromodulation strategies.

## Introduction

Brain stimulation has become a central neuromodulation strategy in both basic research and clinical practice (Davidson et al., 2024). A core challenge is how to dynamically monitor the activity of the brain regions engaged by stimulation, so that the evoked neural responses and their network dynamics can be evaluated as they unfold (Soleimani et al., 2023). In clinical settings, local electrophysiology already supports closed-loop brain stimulation in selected indications, but its recordings are spatially confined and do not directly resolve the stimulation-evoked dynamics that unfold across broader circuits (Herron et al., 2026). A spatially resolved, network-level readout could complement the locally recorded electrophysiology that is predominantly used in closed-loop brain stimulation and support the discovery of circuit-level biomarkers. Without such a readout, it is difficult to dissect the mechanisms of brain stimulation or to optimize stimulation parameters. Each of the established functional imaging modalities, however, involves different trade-offs in spatiotemporal resolution, depth coverage, and experimental flexibility (McFadyen and Dolan, 2023).

Functional ultrasound (fUS) imaging, built on ultrafast plane-wave acquisition (Macé et al., 2011), has emerged as a promising approach for monitoring these network-level responses. It images cerebral blood volume (CBV) changes at the microvascular level across both cortical and subcortical regions, with a spatial resolution of approximately 100 μm and a temporal resolution on the order of hundreds of milliseconds (Montaldo et al., 2022). Among widely used neuroimaging modalities, fUS therefore offers a distinctive combination of spatial resolution, temporal resolution, and imaging depth, contributing to its increasing adoption in neuroscience research in recent years. Electroencephalography (EEG) provides a direct electrophysiological readout with millisecond temporal resolution but has limited spatial localization and is most sensitive to cortical sources. Functional magnetic resonance imaging (fMRI) offers noninvasive whole-brain coverage and strong translational relevance across species but operates on the seconds timescale and relies on bulky scanners that constrain experimental flexibility (McFadyen and Dolan, 2023). Optical methods such as two-photon microscopy and wide-field calcium imaging deliver cellular-level resolution but have limited penetration beyond the superficial cortex (Yang and Yuste, 2017; Grienberger et al., 2022). Miniaturized fUS probes, by contrast, can be head-mounted on awake mice and used for functional imaging in freely moving animals (Urban et al., 2015; Tiran et al., 2017; Bergel et al., 2020), avoiding the anesthesia confound that fMRI rodent studies typically incur. With this profile, fUS has been applied in pharmacological studies (Rabut et al., 2020; Vidal et al., 2020, 2022), disease models (Hingot et al., 2020; Brunner et al., 2023, 2024), and brain–machine interfaces (BMIs) (Norman et al., 2021; Griggs et al., 2024) in preclinical models. In humans, chronic transcranial imaging remains challenging due to skull attenuation and aberration and potential motion artifacts, as well as the seconds-scale latency of neurovascular coupling, which we return to later. Despite this, fUS has been effectively used in several domains where these barriers are less limiting, including intraoperative mapping (Imbault et al., 2017; Soloukey et al., 2020, 2023) and bedside monitoring of neonatal brain function (Demené et al., 2017, 2019; Baranger et al., 2021; Faure et al., 2026).

**Figure 1:**
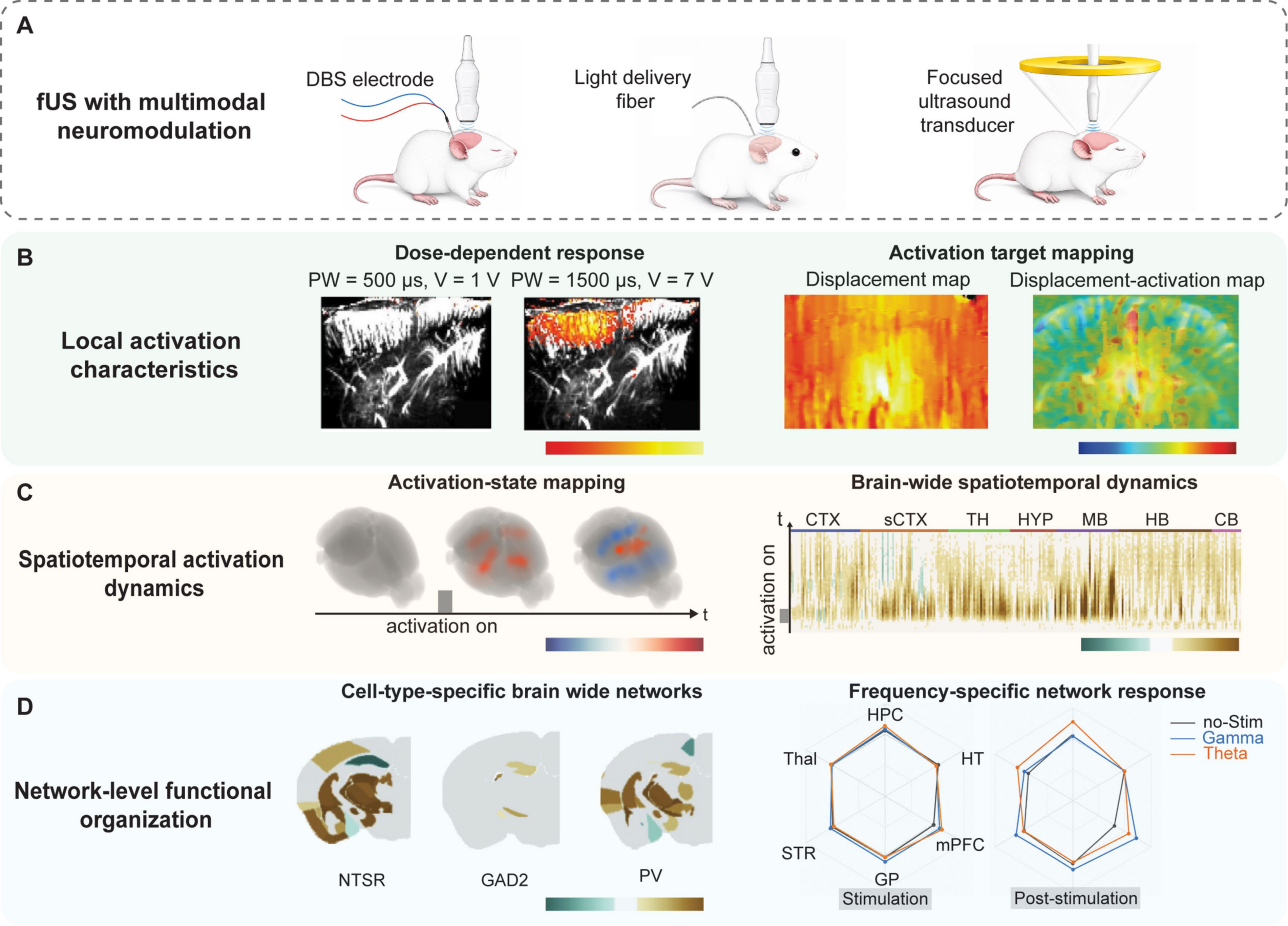
Representative examples of fUS readouts for multimodal neuromodulation. **(A)** Schematic overview of three representative fUS-neuromodulation paradigms, including DBS-fUS, optogenetics-fUS, and FUS-fUS. **(B–D)** Representative examples of fUS readouts at different organizational scales, including local activation characteristics, spatiotemporal activation dynamics, and network-level functional organization. Schematics in **(A)**, left and center, were created by the authors based on Nayak et al. (2021)and Sans-Dublanc et al. (2021), respectively. The schematic in **(A)**, right, was adapted with permission from Murphy et al. (2024), © 2024 Elsevier. Panel **(B)**, left, adapted from Nayak et al. (2021), licensed under CC BY 4.0. Panel **(B)**, right, adapted with permission from Kim et al. (2024), © 2024 Elsevier. Panels **(C)**, right, and **(D)**, left, were adapted with permission from Sans-Dublanc et al. (2021), © 2021 Elsevier. Panel **(D)**, right, adapted from Crown et al. (2024), licensed under CC BY 4.0. CTX, cortex; sCTX, subcortex; TH/Thal, thalamus; HYP/HT, hypothalamus; MB, midbrain; HB, hindbrain; CB, cerebellum; NTSR, neurotensin receptor-expressing neurons; GAD2, glutamate decarboxylase 2-expressing neurons; PV, parvalbumin-expressing neurons; HPC, hippocampus; STR, striatum; GP, globus pallidus; mPFC, medial prefrontal cortex.

Despite this momentum, the application of fUS to brain stimulation has not been systematically reviewed. Existing studies are scattered across stimulation modalities and animal models, with no integrated synthesis to date. This minireview first outlines the imaging principles of fUS, then reviews its application across three representative stimulation modalities: optogenetics, deep brain stimulation (DBS), and focused ultrasound (FUS), followed by the direction we consider most distinctive: an integrated fUS–transcranial FUS (tFUS) closed-loop neuromodulation system, made possible because both readout and stimulation share ultrasound as their physical basis. Figure 1 summarizes the three stimulation–fUS paradigms reviewed here and representative fUS readouts at the local, spatiotemporal, and network levels. The experimental configurations, brain targets, and stimulation parameters of the reviewed preclinical studies are summarized in Table 1. Finally, we address the technical challenges and near-term research prospect.

### Principles of functional ultrasound as a brain readout

The development of fUS depends on advances in ultrafast ultrasound imaging (Bercoff et al., 2011). Conventional B-mode ultrasound uses line-by-line focused scanning: each transmission is focused on a single acoustic line, and the image is built up sequentially (Hangiandreou, 2003). This concentrates acoustic energy and gives high single-line SNR and lateral resolution (Zander et al., 2020), but the frame rate sits at tens of frames per second. Sensitive detection of blood motion requires a high temporal sampling rate, but because line-by-line scanning revisits each location only after sweeping the entire field of view, it yields only sparse temporal samples per pixel (Macé et al., 2011).

Ultrafast ultrasound replaces line-by-line scanning with plane-wave transmission. All array elements fire simultaneously and the entire field of view is imaged in one pulse, so that a complete image is reconstructed from a single transmit–receive event (Montaldo et al., 2009). The raw frame rate jumps from tens of Hz to 1,000–10,000 Hz (Tanter and Fink, 2014), allowing each pixel to be densely sampled over time. The trade-off is that a single plane-wave shot has lower SNR than a focused transmission, resulting in worse image quality (Cikes et al., 2014). This is recovered by coherent compounding: transmitting plane waves from several angles and coherently summing the resulting images. A small number of compounding angles is enough to match the image quality of conventional focused scanning while keeping a frame rate orders of magnitude higher (Montaldo et al., 2009).

The high frame rate does not by itself measure blood flow, but it enables ultrafast power Doppler (Macé et al., 2013). Each pixel now carries a temporally dense echo sequence containing both strong signals from stationary tissue and weaker signals from moving blood; the tissue echoes are typically tens of dB stronger and would otherwise drown out the blood-flow signal (Demené et al., 2015). Spatiotemporal filtering separates the two, typically using SVD-based clutter rejection that exploits their differing statistics (Demené et al., 2015). Integrating the power of the filtered signal over a short time window yields a power Doppler image (Demené et al., 2014), whose intensity is used as a proxy for relative local CBV. The dense temporal samples provided by ultrafast acquisition enable more effective clutter suppression and greatly improve sensitivity to slow microcirculation compared with conventional power Doppler, allowing the resolution of sub-millimeter microvessels that conventional Doppler cannot detect (Aziz et al., 2022).

fUS builds on ultrafast power Doppler. Its link to neural activity relies on neurovascular coupling: local neuronal activity induces changes in vascular tone and CBV, which are detected as power Doppler signal changes (Iadecola, 2017). fUS does not record neuronal electrical activity directly. Instead, it tracks CBV changes through continuous power Doppler imaging and uses them as a surrogate readout of neural activity (Deffieux et al., 2021). A recent simultaneous fUS–Neuropixels study in awake mice showed that fUS amplitude is linearly related to local spike-rate changes, with an estimated spatial spread of approximately 300 μm relative to the spiking signal (Lambert et al., 2025). This supports fUS as a reliable proxy at the local-population level. The temporal trade-off is that each power Doppler frame integrates over hundreds of milliseconds, so the dynamics fUS can resolve sit on the sub-second-to-second scale rather than the millisecond range of action potentials (Macé et al., 2011). For brain stimulation studies, this is well matched to the typical timescale of stimulation-evoked CBV transients, and a sub-second acquisition window is fast enough to support online assessment of stimulation effects.

### fUS readout of optogenetically driven brain circuits

The combination of optogenetics and fUS is naturally complementary: optogenetics specifies the activated cell type, while fUS captures whole-brain hemodynamic responses on a sub-second timescale. Several studies now illustrate distinct uses of this pairing. Brunner et al. (2020) built a 2D-matrix array platform for brain-wide volumetric fUS (vfUS) in awake mice and established a combined optogenetic and vfUSI approach to map cell-type-specific and circuit-level dynamics. By optogenetically stimulating defined neuronal populations in the superior colliculus (SC), they demonstrated both overlapping and distinct activation profiles across downstream subcortical and cortical networks. This framework potentially enables high-resolution, longitudinal whole-brain functional mapping in behaving animals. Sans-Dublanc et al. (2021), also working in awake mice, used optogenetic activation of four collicular cell types in the SC and found that each drove a distinct, partially overlapping whole-brain activation network. Voxel-wise and region-based analyses identified the posterior paralaminar nuclei of the thalamus (PPnT) as a key node, and causal experiments validated its role in suppressing habituation of defensive behavior. Edelman et al. (2021) compared fUS and fMRI under an identical anesthetic protocol in the same Thy1-ChR2 mice during optogenetic stimulation of the primary motor cortex (M1). They found that fUS detected stronger and more widespread CBV-related responses than BOLD fMRI, particularly under low stimulation intensities, demonstrating the superior sensitivity of fUS in capturing optogenetically evoked neurovascular responses. Provansal et al. (2021) used the red-shifted opsin ChrimsonR in anesthetized rats to detect optogenetic activation in the deep layers of the primary visual cortex (V1). They mapped the spread of V1-initiated activity to the lateral geniculate nucleus (LGN) and the secondary visual cortex (V2), with electrophysiological recordings confirming the neuronal origin of the fUS-detected CBV changes.

Opto-fUS experiments face several confounders that need careful control. First, light exposure itself can lower intracellular calcium in arterial smooth muscle cells, producing vasodilation and CBV increases unrelated to neuronal activity (Rungta et al., 2017). Without wavelength-matched, opsin-free controls, this light-only effect can be mistaken for a stimulation response. Second, the hemodynamic response can adapt across repeated stimulation rounds, such that it is possible to observe decreases over consecutive epochs (Brunner et al., 2020). Third, evidence from other optogenetic hemodynamic imaging modalities indicates that anesthesia can attenuate network recruitment or even alter response polarity, depending on the targeted cell type and anesthetic regimen (Desai et al., 2011; Lee et al., 2021; Hamada et al., 2024). Anesthetic conditions should therefore be carefully standardized and reported, and findings obtained under anesthesia should not be directly generalized to awake animals. These caveats differ in kind: light-induced vasodilation is a modality-related artifact, whereas adaptation and anesthesia-dependent responses reflect genuine but state-dependent biological processes. Together, they caution against interpreting opto-fUS responses as a one-to-one proxy for neuronal activity without appropriate controls. Despite these limitations, opto-fUS remains the most developed of the stimulation-fUS combinations and a powerful tool for mapping *in vivo* circuits involving genetically defined neuronal populations (Brunner et al., 2020), with natural next steps in awake behavior, 3D acquisition, and more cell-type-specific opsins.

**Table 1:** Preclinical fUS studies of brain stimulation.

Stimulation modality	Awake	fUS configuration	Imaging plane	Brain target	Stimulation parameters
Optogenetics (Brunner et al., 2020)	Yes	Vantage 256; 1,024 elements; 14.5 MHz^a^	Volumetric	sSC; GRP/NTSR/GAD2-Cre; ChR2	473 nm^b^; 1.5 mW/mm^2^^c^; 2 ms × 20 Hz × 2 s^d^
Optogenetics (Sans-Dublanc et al., 2021)	Yes	Vantage 128; 128 elements; 15 MHz	Sagittal	sSC; CaMKII-AAV/NTSR/PV/GAD2-Cre; ChR2	473 nm; 9.5–12.5 mW/mm^2^; 2 ms × 20 Hz × 1 s, 2 ms × 50 Hz × 1 s, 2 ms × 5 Hz × 4 s
Optogenetics (Provansal et al., 2021)	No	Aixplorer; 128 elements; 15 MHz	Coronal	V1; CaMKII-AAV; ChrimsonR	595 nm; 140 mW/mm^2^; 25 ms × 20 Hz × 2 s
Optogenetics (Edelman et al., 2021)	No	Vantage 128; 128 elements; 15 MHz	Coronal	M1; Thy1; ChR2	473 nm; 0.8–8 mW/mm^2^; 20 ms × 10 Hz × 12 s
DBS (Nayak et al., 2021)	No	Vantage 256; 128 elements; 18.5 MHz	Sagittal	VL thalamus	1–7 V; 50–1,500 μs × 10 Hz/100 Hz × 10 s
DBS (Crown et al., 2024)	No	Iconeus One; 128 elements; 15 MHz	Sagittal	MSN	80 μA; 100 μ s^e^ × 7.7 Hz × 5 min, 100 μ s × 100 Hz × 5 min
FUS (Kim et al., 2024)	No	Vantage 256; 128 elements; 15.625 MHz	Coronal	Sensorimotor cortex and subcortical regions	4 MHz^f^, 1 kHz AM^g^; 23–360 W/cm^2^^h^; 150/300 ms × 1 Hz × 1/5/10 s
FUS (Murphy et al., 2024)	No	Vantage 256; 128 elements; 15 MHz	Coronal	Central medial thalamus	500 kHz; 18.9 W/cm^2^; 80 ms × 2.5 Hz × 5 s

a fUS imaging-transducer center frequency.

b Optogenetic stimulation wavelength.

c Optical irradiance, as reported.

d Single-pulse duration × pulse repetition frequency × train/sonication duration.

e The original article lists 100 ms; 100 μs is reported here based on the cited protocol (Zepeda et al., 2022) and the temporal constraints of 100-Hz stimulation.

f FUS carrier or fundamental frequency, as reported in the original study.

g Amplitude modulation frequency.

h FUS stimulation intensity, reported as spatial-peak pulse-average intensity (*I*_SPPA_).

### DBS–fUS for parameter-dependent network mapping

DBS delivers electrical pulses through implanted electrodes to modulate activity (Krauss et al., 2021). In both research and clinical practice, DBS outcomes depend strongly on stimulation parameters such as frequency, amplitude, pulse width, and target location, and these parameters are largely selected through trial and error. fUS provides a near-real-time, network-level readout of how each parameter combination engages local and distal hemodynamic responses, with enough spatial resolution to distinguish nearby nuclei and enough coverage to track effects beyond the stimulation site.

Two preclinical fUS studies illustrate this approach. Nayak et al. (2021) stimulated the ventrolateral thalamus (VL) in rats and used fUS to monitor CBV changes in the M1. They found a strong frequency-by-amplitude interaction: at high voltages (5–7 V), low-frequency stimulation (10 Hz) drove markedly stronger M1 activation than high-frequency stimulation (100 Hz), whereas high-frequency stimulation was more efficacious at low intensities. The data make clear that DBS effects on motor cortex cannot be predicted from frequency or amplitude alone, and that fUS can quantify how the two parameters interact.

Crown et al. (2024) extended this approach to cognition-related circuits. In an MK-801 mouse model of reduced NMDA receptor function, they applied DBS to the medial septal nucleus (MSN) and tracked CBV across the septohippocampal network. Theta-frequency stimulation (7.7 Hz) enhanced hippocampal CBV, and the effect persisted after stimulation ended; high-frequency stimulation (100 Hz) produced no comparable response. The post-stimulation persistence matters because lasting modulation is often what clinical DBS aims for, and fUS can track whether and where it occurs.

Only two pioneering studies have been reported to date, largely reflecting the substantial technical challenges associated with combining DBS and fUS. The metallic electrodes typically used for DBS have a large acoustic impedance mismatch with brain tissue, producing strong specular reflections and shadowing artifacts in ultrafast power Doppler images, which obscure hemodynamic signals near the electrode and along its acoustic shadow. At the same time, DBS can recruit distributed brain networks, making volumetric imaging important for comprehensively mapping stimulation-evoked responses. Existing DBS–fUS studies have relied on linear-array imaging of selected 2D planes, for example by positioning the imaging plane contralateral to the implanted electrode to reduce direct interference (Crown et al., 2024). Although this strategy can mitigate electrode-related artifacts, it excludes ipsilateral and out-of-plane regions and therefore limits brain-wide circuit mapping. Three-dimensional acquisition could address the incomplete spatial coverage but would not, by itself, eliminate electrode-related artifacts. Mitigating these artifacts remains a more difficult challenge, and future progress may come from non-metallic electrode designs (Panskus et al., 2026) as well as new image-reconstruction approaches that explicitly account for the artifact pattern.

### From fUS imaging of FUS effects to closed-loop fUS–tFUS neuromodulation

Like DBS, FUS neuromodulation has a large parameter space, including frequency, intensity, pulse repetition frequency, duty cycle, and focal location, and the network-level effects can extend well beyond the focal spot. fUS offers a way to characterize and optimize this parameter space through hemodynamic readouts. Two recent studies illustrate this. Kim et al. (2024) combined fUS with a fully ultrasound-based *in situ* targeting system, performing dose-dependent characterization of FUS parameters and using displacement-activation maps to establish the spatial correspondence between tissue displacement and CBV changes. Murphy et al. (2024) systematically mapped the parameter windows for FUS-induced excitation and inhibition and used fUS to track the spatial distribution of post-stimulation hemodynamic effects, revealing widespread reductions in CBV following FUS stimulation.

It should be noted that thermal effects induced by FUS may influence vascular responses, and therefore fUS signals in FUS experiments require careful interpretation (Kim et al., 2024). In addition, cranial preparation and coupling conditions may also alter cortical temperature. An open craniotomy can cool the exposed cortical surface (Kalmbach and Waters, 2012), and a temperature reduction of approximately 2–3 °C during cranial-window imaging has been shown to decrease resting capillary blood flow (Roche et al., 2019). These temperature-related effects should therefore be considered when interpreting regional fUS responses.

Among the stimulation–fUS combinations covered above, fUS–tFUS has a structural advantage that the others do not have: both the readout and stimulation components rely on the same physical basis and can share a similar hardware ecosystem and signal processing pipelines. The stimulation–imaging loop can, in principle, be closed on a single ultrasound system, with fUS providing an online readout of depth-resolved brain hemodynamic states and a controller using this information to dynamically adjust the parameters, target location, and timing of tFUS. Both components of this potential read–write framework have begun to show technical feasibility. On the readout side, a recent fUS brain–machine interface study demonstrated single-trial decoding of motor plans in non-human primates, with decoder performance maintained across sessions separated by days to months after image-registration-based alignment (Griggs et al., 2024). On the write side, low-intensity tFUS has shown promise for noninvasive modulation of cortical and deep brain targets (Kim et al., 2021). These advances suggest that an integrated tFUS–fUS framework may be technically feasible, although further validation is needed before it can be considered a mature closed-loop neuromodulation platform.

Proof-of-concept studies have begun to establish this read–write architecture. As discussed above, fUS can read out tFUS-evoked cerebral hemodynamic changes, demonstrating that ultrasound-based stimulation and functional imaging can coexist within the same animal and experimental paradigm (Kim et al., 2024; Bendig et al., 2025). The next step is to close the control loop, in which the imaging readout would be used to adapt stimulation parameters. To date, closed-loop ultrasound neuromodulation studies have relied mainly on EEG feedback (Zhong et al., 2021). Although EEG provides fast neural readouts, its spatial sensitivity is weighted toward cortical-surface activity, limiting its ability to resolve deep circuit dynamics. fUS-based feedback could therefore extend closed-loop control to subcortical and network-level responses, with potential relevance to seizure foci in the mesial temporal lobe, basal-ganglia circuits in Parkinson’s disease, and thalamocortical loops in chronic pain.

Two challenges remain. The first is transcranial acoustic attenuation. The skull absorbs, scatters, and distorts acoustic waves on both transmit and receive paths, degrading fUS image quality and effective stimulation focality in models beyond young rodents. Several complementary directions are currently under active development: acoustically transparent cranial window implants (Rabut et al., 2024), chemically cleared skull approaches (Wang et al., 2025; Landry et al., 2026; Ramezanpour et al., 2026), and acoustically engineered metamaterials (Li et al., 2022; Dong et al., 2025), which seek to mitigate the problem at the cranial interface, biological, and materials levels. Given the diversity of cranial preparations and fUS acquisition and processing pipelines, standardized reporting of these parameters will also be important for reproducibility and cross-laboratory comparison. The second is the temporal delay of the neurovascular coupling signal. Because fUS reads CBV rather than electrical activity, fUS-based closed-loop control cannot operate at the millisecond timescale supported by EEG or local electrophysiology. This limits its suitability for applications requiring rapid detection of electrical events or phase-locked intervention, such as oscillation-phase-locked stimulation (Zrenner et al., 2018; Schaworonkow et al., 2019). However, this is not a fundamental obstacle for many compelling applications that do not require sub-second control, such as pharmacological monitoring, bedside brain-state monitoring, and brain–machine interfaces. For these applications, the controlled variables evolve on timescales that match hemodynamic feedback, and the seconds-scale latency of neurovascular coupling is therefore not necessarily a limiting factor.

fUS and tFUS represent a distinctive pairing in neuroengineering, combining ultrasound-based functional readout with ultrasound-based stimulation. It is important to note, however, that most available evidence remains preclinical and is derived primarily from animal models. In addition to the challenges discussed above, issues related to safety constraints and real-time processing requirements must also be addressed before this approach can be translated into routine clinical practice. As skull-compensation methods, stimulation protocols, and signal processing pipelines continue to mature, this combination could move from proof-of-concept toward clinically relevant implementation.

## Discussion

fUS has emerged as a working readout platform for brain stimulation. Across the optogenetic, DBS, and FUS paradigms reviewed here, it can resolve cell-type-specific network responses, parameter-dependent activation patterns, and stimulation-induced hemodynamic effects, often within the same animal and the same session. This combination of depth access, spatiotemporal resolution, and experimental flexibility is difficult to achieve with most widely available neuroimaging modalities. A particularly distinctive trajectory is a closed-loop fUS–tFUS system, in which ultrasound-based readout and ultrasound-based stimulation are integrated within a shared physical and hardware framework. Although both fUS readout and tFUS stimulation have been demonstrated separately, closing the loop will require not only hardware integration but also latency-aware state estimation, artifact-robust acquisition, safety constraints, and validation of state-dependent stimulation rules. Near-term targets may include seizure suppression, where pre-ictal or ictal hemodynamic changes could provide candidate control signals. Plausible longer-term targets include closed-loop modulation of basal-ganglia circuits in Parkinson’s disease and thalamocortical loops in chronic pain.

Beyond the stimulation modalities reviewed here, the pairing of fUS with TMS and tDCS remains largely absent from the literature, in part because of spatial-geometry constraints, electromagnetic interference, and the difficulty of maintaining stable acoustic access during noninvasive stimulation. Nevertheless, following stimulation, fUS could complement these modalities by providing a mesoscale hemodynamic readout of network engagement. This perspective aligns with the broader move toward biomarker-oriented neuromodulation, in which measures of cortical excitability, stimulation-induced plasticity, and individual responsiveness are increasingly used to guide personalized stimulation strategies (Gogulski et al., 2023; Carè et al., 2024; Fitzsimmons et al., 2024). TMS-derived neurophysiological measures, such as motor-evoked potential amplitudes, can provide candidate indices of corticospinal excitability and its modulation following stimulation (Di Fazio and Palermo, 2026; Di Fazio et al., 2026), whereas fUS could complement these measures by adding spatially resolved information about the downstream circuits and vascular-network states engaged by stimulation. Importantly, combining fUS with electrophysiological measures can provide a more comprehensive characterization of neural activity and its associated hemodynamic responses. At the physiological level, the electrical activity and vascular responses involved in neurovascular coupling are not linked in a one-to-one manner, making it difficult for a single modality to distinguish changes in neural activity from alterations in neurovascular coupling. Moreover, this combination provides complementary spatiotemporal information, and their combined use can reveal additional pathophysiological features (Zhang et al., 2024) and resolve coupling patterns associated with distinct neuronal populations (Landemard et al., 2026), thereby supporting precision neuromodulation. Across these directions, what fUS enables is both a more integrated, multimodal characterization of stimulation-induced brain responses and a shift in how neuromodulation is run, from observing outcomes after stimulation to reading brain states during stimulation and updating stimulation strategies in response.
